# Land-use and land-management change: relationships with earthworm and fungi communities and soil structural properties

**DOI:** 10.1186/1472-6785-13-46

**Published:** 2013-12-01

**Authors:** David J Spurgeon, Aidan M Keith, Olaf Schmidt, Dennis R Lammertsma, Jack H Faber

**Affiliations:** 1 Centre for Ecology and Hydrology, Maclean Building, Benson Lane, Crowmarsh Gifford, Wallingford, Oxon OX10 8BB, UK; 2 Centre for Ecology and Hydrology, Library Avenue, Bailrigg, Lancaster LA1 4AP, UK; 3 UCD School of Agriculture and Food Science, University College Dublin, Dublin 4, Belfield, Ireland; 4 Alterra, Wageningen University and Research Centre, P.O. Box 47, 6700 AA Wageningen, Netherlands

**Keywords:** Meta analysis, Earthworm, Fungi, Functional biodiversity, Soil porosity, Microaggregate stability

## Abstract

**Background:**

Change in land use and management can impact massively on soil ecosystems. Ecosystem engineers and other functional biodiversity in soils can be influenced directly by such change and this in turn can affect key soil functions. Here, we employ meta-analysis to provide a quantitative assessment of the effects of changes in land use and land management across a range of successional/extensification transitions (conventional arable → no or reduced tillage → grassland → wooded land) on community metrics for two functionally important soil taxa, earthworms and fungi. An analysis of the relationships between community change and soil structural properties was also included.

**Results:**

Meta-analysis highlighted a consistent trend of increased earthworm and fungal community abundances and complexity following transitions to lower intensity and later successional land uses. The greatest changes were seen for early stage transitions, such as introduction of reduced tillage regimes and conversion to grassland from arable land. Not all changes, however, result in positive effects on the assessed community metrics. For example, whether woodland conversion positively or negatively affects community size and complexity depends on woodland type and, potentially, the changes in soil properties, such as pH, that may occur during conversion. Alterations in soil communities tended to facilitate subsequent changes in soil structure and hydrology. For example, increasing earthworm abundances and functional group composition were shown to be positively correlated with water infiltration rate (dependent on tillage regime and habitat characteristics); while positive changes in fungal biomass measures were positively associated with soil microaggregate stability.

**Conclusions:**

These findings raise the potential to manage landscapes to increase ecosystem service provision from soil biota in relation to regulation of soil structure and water flow.

## Background

National monitoring programmes have often identified that soils under different land use and land management regimes harbour differing soil communities [1,2]. In a limited number of cases the primary drivers of this variation have been identified, such as the strong influence of soil pH on bacterial communities [3]. When augmented by the results of smaller scale experimental and monitoring studies, which have frequently shown similar effects, these observations suggest a strong forcing effect of incumbent land use on overall soil community size and composition. Such community changes can have implications for soil functioning. In particular, the activities of specific groups of the soil biota (e.g. microorganisms, ecosystem engineers) are considered important to many soil functions that underpin the provision of ecosystem goods and services. These functionally relevant groups have become, therefore, an established focus for soil community and ecosystem process research [4-8].

With a relative wealth of data available describing patterns of land use associated change in overall abundance (at least for well-studied taxa) and also studies that link soil community characteristics to ecological metrics e.g. [3,6,9-11], quantifying these community effects and establishing their functional links to soil processes is potentially achievable. Here we have sought to summarise how some of the best-studied changes in land use and management affect community metrics for two key functional components of the soil biota, namely earthworms and soil fungi. We focus on transitions of extensification in land use that may benefit these taxa. Further, we relate community size changes for these two groups to influences on soil structural properties, thereby attempting to quantify implications of land use changes to functional linkages in the soil ecosystem.

The approach we have chosen for the assessment was based on a systematic review and meta-analysis of the published literature. For earthworms, the analysis focussed on assessing the effects of land use and land management changes on overall community size. This focus on total abundance was chosen because in published studies, detailed community characteristics, such as species identity, were often not reported; and this would have limited the size of the available data-set. For soil fungi, both overall abundance measures and community structural indicators were considered. This reflects the wider variety of metrics reported in the literature for this group. For both taxa, we sought to quantify the magnitude of community change occurring when land management practices or land use convert from more intensive to less intensive conditions. Three such scenarios were considered 1) implementation of reduced tillage regimes to previously intensively tilled lands; 2) conversion of managed arable land to natural or managed grassland; 3) successional conversion or afforestation of grassland to woodland.

To extend our review, we also considered the implications of earthworm and fungal community changes for soil properties in two further literature analyses. For earthworms, because the multiple roles of this group on greenhouse gas emissions and carbon sequestration have recently been comprehensively appraised [12], we focussed instead on the effects of community size and functional group (epigeic, endogeic, anecic) composition on soil porosity. This recognises the role of earthworms as ‘ecosystems engineers’ , capable of altering their physical environment through their casting and burrowing activities [13,14]. For soil fungi, the association of community measures with soil microaggregate stability was determined to investigate the potentially important role of this group in the formation of soil aggregates, via hyphal growth and the production of coagulating substances like glomalin [15,16]. These two analyses, thus, both consider how changes in community metrics for these two groups can translate through to effects on soil structure.

## Results

### Earthworm communities

#### Arable to grassland conversion

From an initial 1466 potential papers identified by the search terms, 16 articles containing 54 data-sets reported earthworm population size and associated variances in regional plots under both conventional arable and grassland land uses (see Table 1 for number of papers and data-sets identified for each assessment, and Additional file 1 for meta-data summaries and Additional file 2 for detailed information). Across all studies, average earthworm abundance (± SD) was 56.3 ± 70.8/m^2^ under arable and 229 ± 193/m^2^ under grassland. Overall the effect size following arable to grassland conversion was significantly positive (ES = 1.178 ± 3.85 95% CI, p < 0.001). This highlights a strong beneficial effect of grassland conversion on earthworms. Vote counting indicated that 48 (89%) of the data pairs showed higher community size under grassland and 6 (11%) higher abundance in arable plots.

**Table 1:** Number of papers containing data and paired data-sets identified for the initial systematic reviews conducted for earthworm and fungal community metrics following land use transition and earthworm effect on water infiltration rates and fungal effects on microaggregate stability

		**Earthworms**	**Fungi**
Conventional vs reduced tillage	Papers	79	15
	Datasets	162	73
Arable to grassland conversion	Papers	16	24
	Datasets	54	173
Grassland to woodland conversion	Papers	15	18
	Datasets	33	85
Earthworm abundance and water infiltration	Papers	5	N/A
	Datasets	29	N/A
Fungal biomass and microaggregate stability	Papers	N/A	10
	Datasets	N/A	86

**Figure 1:**
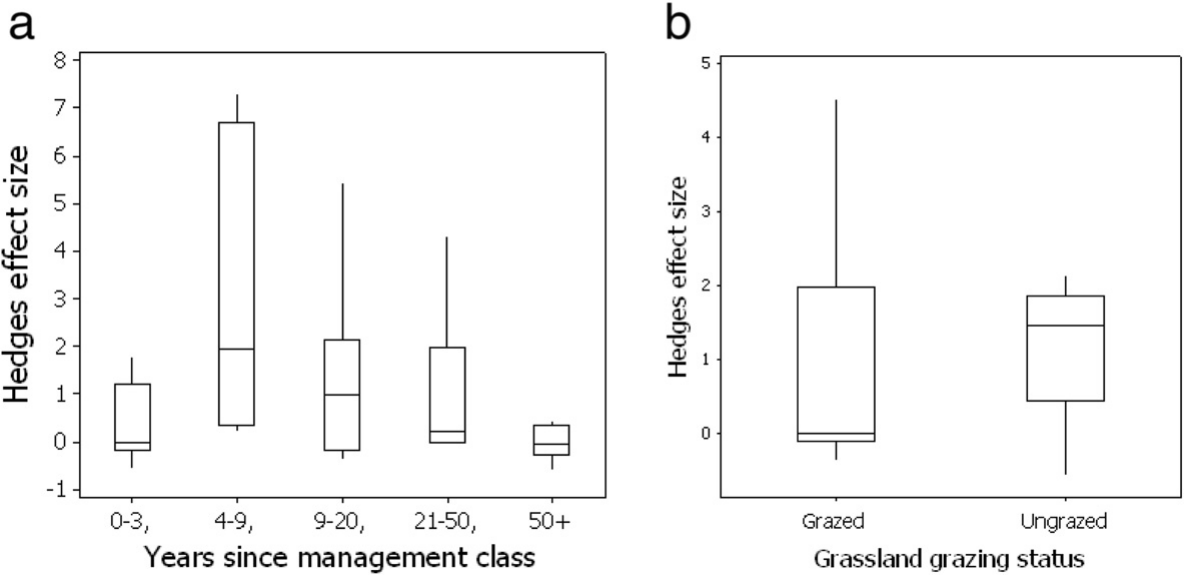
**Box plot of the effect size of earthworm population change in paired arable and transitioned grassland plots classified to (a) categories based on time elapsed since conversion from arable to grassland and (b) grazing status of the converted grassland.** Boxes indicate lower and upper quartile values, mid line the geometric mean and whiskers the 95% confidence intervals.

A key variable that may influence earthworm community size following conversion to grassland is the time elapsed since change. Fitting a linear model did not, however, suggest there was a significant relationship between time elapsed and effect size (p > 0.05, r^2^ = 0.01). Categorisation of studies into ‘age’ classes (years since conversion) indicated that the benefits of conversion to grassland on earthworm abundance were evident even after relatively short-term (0–3 years) durations (Figure 1a). Only marginal abundance benefits then accrue with extended grassland maintenance, and the differences in effects sizes between age classes were not significantly different (GLM F = 1.97, p > 0.05).

A further factor which may influence earthworm abundance following conversion is the intensity of grassland use (e.g. for grazing). Categorisation of grasslands into grazed and ungrazed classes and analysis using a fixed effects model, however, indicated no significant effect of grazing status on the effect size for earthworm abundance change (GLM F = 0.02, p > 0.05) (Figure 1b). This suggests a common response of earthworm population abundance to conversion independent of ultimate grassland use.

#### Grassland to woodland conversion

The search terms identified 212 potential papers of which 60 were selected for detailed analysis based on the selection criteria (see Methods section). Of these, 15 papers containing 33 data-sets provided quantitative information for analysis of earthworm density and variance in paired grassland vs. deciduous, coniferous, tropical or orchard/agroforestry woodland plots. Average grassland population size among these data-sets was 341 ± 402/m^2^ (n.b. this is higher than the value of 222 ± 206/m^2^ found for the grassland plots in the arable conversion review), compared to 315 ± 431/m^2^ for the woodland plots. The effect size following grassland to woodland conversion was slightly, but not significantly, negative (ES = -0.033 ± 2.72 95% CI, P >0.05). Vote count analysis indicated that 19 (58%) data-sets showed larger populations in grasslands, 12 (36%) larger populations in the woodland and 2 (6%) no difference between the two plot types.

**Figure 2:**
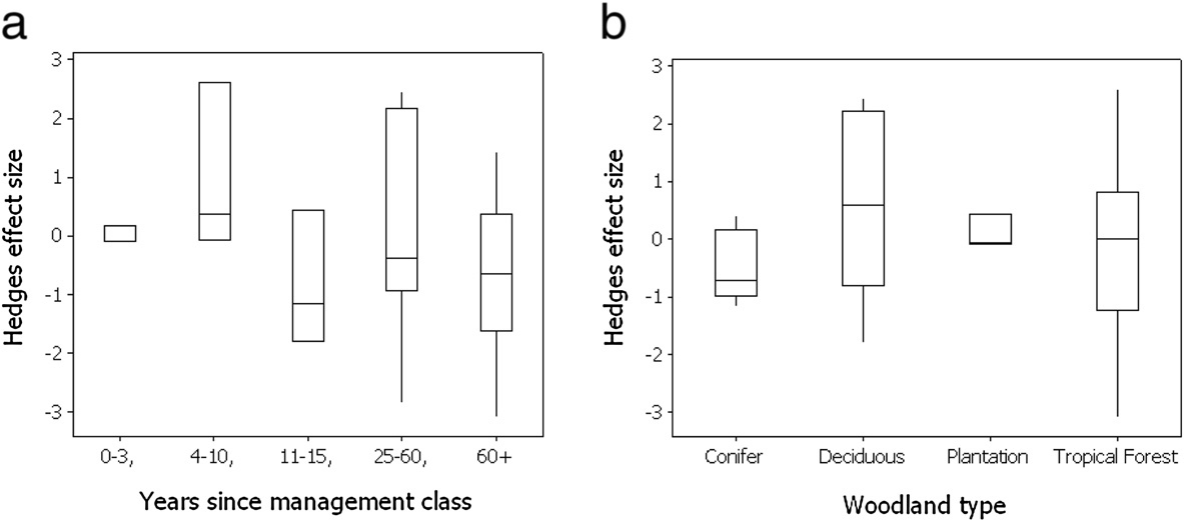
Box plot of the effect size of earthworm population change in paired grassland and transitioned woodland plots classified to (a) categories based on time elapsed since conversion from arable to grassland and (b) woodland types.

Although no overall significant effect on woodland conversion was observed, sub-set analyses were nonetheless conducted to assess how age since transition (i.e. the age of tree stands) and woodland type (temperate deciduous, temperate coniferous, tropical, agroforestry) influence earthworm communities. For time since conversion, a linear model indicated no significant time associated trend for effect size (p > 0.05, R^2^ = 0.01). The absence of an effect of stand age was confirmed in an age class analysis (GLM F = 0.98, p > 0.05) (Figure 2a). The categorisation of studies into four forest types, temperate deciduous, temperate coniferous, tropical, and orchard/agroforestry indicated no significant influence of forest type on effect size (GLM F = 1.72, p > 0.05). The strongest reductions in abundance were seen following pasture conversion to coniferous forests compared to conversion to other woodland systems (Figure 2b). As it is known that plant-derived inputs can reduce pH, the effect of pH shift following conversion to woodland on population metrics was further investigated. Although the largest reductions were associated with the largest pH shifts, in a linear model the pH change relationship with effect size was not significant (y = 331 + 0.285×, F = 1.54, p > 0.05, R^2^ = 0.12).

**Figure 3:**
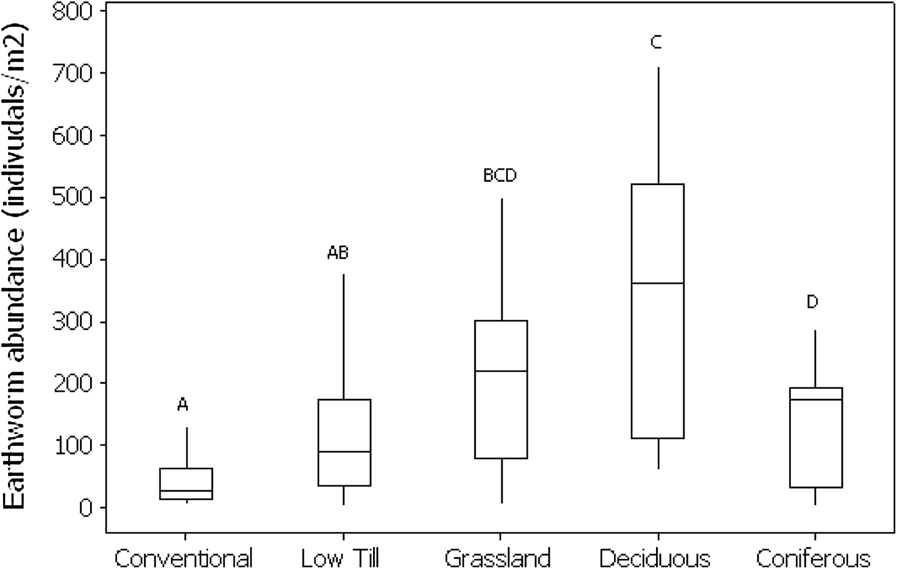
**Box plot of earthworm abundance under a series of habitat types (nb data for grassland come from the Arable to grassland conversion study).** Boxes indicate lower and upper quartile values, mid line the geometric mean and whiskers the 95% confidence intervals. Treatment not sharing the same letter are significantly different at p < 0.05, according to Tukey’s post-hoc test.

In the context of land management and land use, the analysed comparisons represent a clear succession and land use intensity gradient from conventionally managed arable fields to mature woodlands. Bringing results together across all studied land uses, a generally positive relationship of earthworm abundance with successional change and/or reducing land use intensity was observed. This was characterised by an increased abundnace in the order conventional arable < reduced till arable < grassland ≤ woodland (except coniferous). This effect of land use on earthworm abundance was highly significant (GLM F = 12.99, p < 0.001), with population abundance significantly higher in grassland and both woodland types than arable land (Figure 3). Noticeably, relatively large increases in earthworm abundance occur even when the land-use intensity change is relatively modest. Thus, reduced till management provides almost half of the benefit associated with full grassland and indeed woodland conversion in terms of the resulting increase in earthworm numbers even though, in this case, the primary land use (i.e. arable) is retained.

### Soil fungal populations and communities

The soil fungal data were richer and more variable in studied metrics than the earthworm data-sets, introducing an additional level of complexity (see Additional file 3). Fungal community metrics that were reported included endpoints as diverse as colony-forming units (CFU), spore density and diversity, plant root-length colonized by mycorrhiza, ectomycorrhizal root tips, glomalin-related soil protein levels, ergosterol, phospholipid fatty acid (PLFA), neutral lipid fatty acid (NLFA) and glucosamine sugars. For analysis these metrics were categorized into either ‘Biomass’ , ‘Colonisation’ or ‘Diversity’ measures (see Table 2 for numbers of comparisons in each category).

**Table 2:** Number of fungal community data-pairs per land-use transition type categorised by study type and metric type

**Metric**		**Land use transition**			
**Type**	**Subtype**	**Conventional vs reduced till**	**Arable to grassland**	**Grassland to woodland**	**Totals**
**Biomass**					
	Biomass	1	-	8	9
	CFU	3	-	-	3
	DNA	-	6	-	6
	Ergosterol	-	4	12	16
	Glomalin	20	47	5	72
	Hyphae	20	7	4	31
	NLFA	-	2	2	4
	PLFA	5	45	2	52
	Spores	7	24	36	67
**Colonisation**					
	Hyphae	-	4	-	4
	Root length	17	6	10	33
	Root tips	-	-	6	6
**Diversity**					
	CFU	3	-	-	3
	Molecular	-	24	-	24
	PLFA	1	1	-	2
	Spores	2	5	-	7
**Totals**		79	175	85	

#### Conventional to reduced tillage

**Figure 4:**
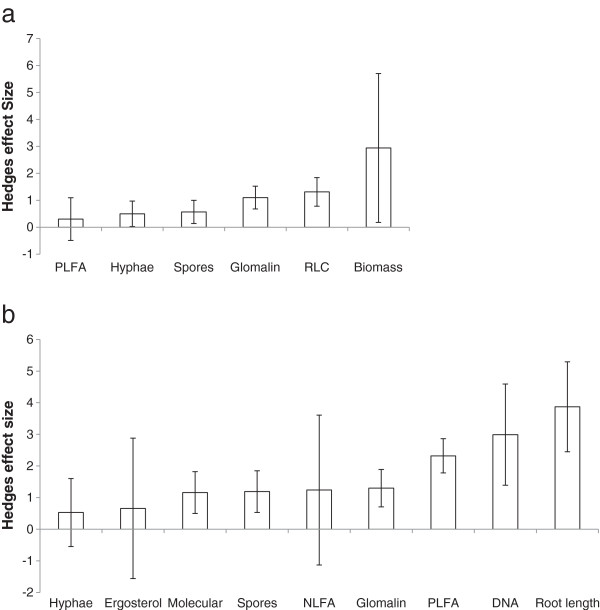
**Effect size for fungal community metrics under (a) paired conventional and reduced tillage arable plots and (b) paired arable and transitioned grassland plots.** Bars represent 95% confidence intervals.

Searches identified 15 papers containing 73 ‘Field’ datasets comparing quantitative changes in fungal communities between conventional and reduced tillage regimes. Overall effect size under reduced or no-till was significantly positive (ES = 0.873 ± 0.243 95% CI, p < 0.001). For stratified analysis, a number of models were tested to examine the sources of heterogeneity within the field data-set range. There were significant differences between the effect sizes of Biomass, Colonisation and Diversity measures (QM = 53.09, P < 0.001), with Colonisation measures having a greater effect size (1.31 ± 0.282 95% CI) compared to Biomass (0.772 ± 0.535 95% CI) and Diversity (0.530 ± 1.14 95% CI). Likewise, significant effect size variation was found between metric subtypes (QM = 49.18, p < 0.001) indicating that in a proportion of studies heterogeneity can be related to the community composition endpoint assessed. Significant positive effects of reduced or no-till transition were found for fungal biomass (P < 0.05), glomalin (P < 0.001), fungal hyphae (P < 0.05) and root length (p < 0.001) but not spore density or PLFA measures (Figure 4a). This suggests these significant measures may provide more sensitive means to detect such effects. There were no significant differences between effect size when reduced and no-till regime studies were compared (QM = 0.102, p > 0.05) for any metric.

#### Arable to grassland conversion

For the arable to grassland conversion study, 24 papers with a total of 173 ‘Field’ datasets were identified. The overall effect size on fungal measures of a conversion from arable to grassland land use was significantly positive (ES = 1.65 ± 0.299 95% CI, p < 0.001). Detailed analysis found no significant differences in the effect sizes resulting from conversion between the three major measure types (QM = 1.536, p > 0.05). An analysis of the different metric sub-types did, however, identify a highly significant effect (QM = 28.615, p < 0.001). This indicated that ergosterol, hyphae or NLFA seemingly did not respond to conversion, whereas DNA (p < 0.001), glomalin (p < 0.001), molecular richness (p < 0.001), PLFA (p < 0.001), root length colonized (p < 0.001) and spore density (p < 0.001) were all enhanced (Figure 4b). Fitting a linear model suggested a significant relationship between time since conversion and effect size of community change (p < 0.001, r^2^ = 0.30). Analysis of the effects of grazing regime (grazed vs. ungrazed) on fungal communities under converted grassland indicated no significant influence on effect size resulting from conversion dependent on grassland use (QM = 0.4820, p > 0.05).

#### Grassland to woodland conversion

**Figure 5:**
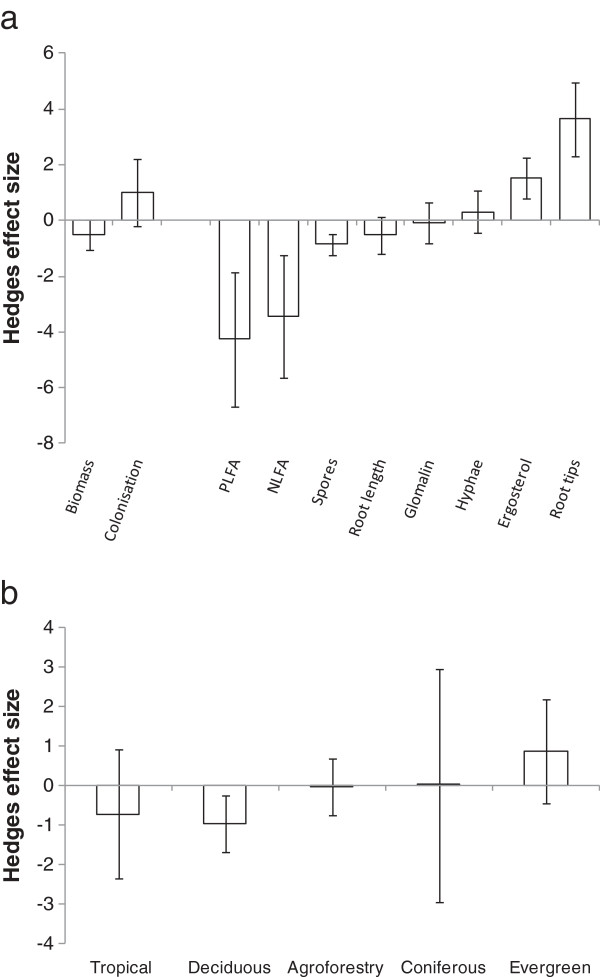
**Effect size for fungal community metrics under (a) paired grassland and woodland plots and (b) for all community parameters between different woodland classes.** Bars represent 95% confidence intervals.

Fungal community responses to grassland conversion to woodland were reported in 18 ‘Field’ datasets. In contrast to the other two land use transitions, the effect size following grassland to woodland conversion was significantly negative (effect size = -0.264 ± 0.485 95% CI, p < 0.001). Differences between the major effect size classes were also significant (QM = 5.283, p < 0.05). The indication was that Biomass measures had a significant negative response, while Colonisation measures had a positive effect size. Such discrpancies could potentially be attributed to a shift from arbuscular mycorrhizal to ectomycorrhizal fungi. This was also largely reflected in the heterogeneity of metric subtypes (QM = 92.1, p < 0.001) with PLFA/NLFA measures being significantly negative (p < 0.0001) and root tip measures significantly positive (p < 0.001) (Figure 5a).

Forest classes showed significant differences for effect size following conversion (QM = 23.622, p < 0.001). On change to deciduous woodland from grassland fungal communities showed a significant negative effect size (p < 0.01) (Figure 5b). No other forest classes showed a significant effect, although this finding should be treated with some caution due to the relatively small sample sizes available for this analysis. An overall negative relationship with fungal measures and time since conversion to woodland was found. This relationship was, however, highly influenced by a few data-points at 50 years since conversion. This may suggest that our analyses may be most relevant to changes in the arbuscular mycorrhizal fungi communities.

### Soil biology and soil structural properties

For earthworm and water infiltration relationships, literature searching yielded 174 potential data sources. Focussing only on temperate biomes, 30 relevant data-sets were identified from 3 separate published studies [17-19] and one unpublished assessment using adult worm data (Faber et al. unpublished results) (see Additional file 4 for details). Analysis of earthworm density and infiltration metrics from these data-sets indicated that earthworm abundance, tillage system and habitat type all had a significant effect on infiltration (GLM, p < 0.01 in all cases). Terms for reduced tillage, grassland land use and average earthworm abundance were all positive and significant within the model, suggesting positive influences in all cases (Table 3). Inclusion of the interaction terms indicated that these were not significant in any case.

**Table 3:** Akaike information criterion and Akaike weights for models to assess relationships between metrics and soil water infiltration rate

**Model types**	**Model terms**	**AlC**	**exp (AlCmin-AlCi)**	**Akaike weight**
1 term models	2	52.978	0	0
	1	58.471	0	0
	4	64.397	0	0
	3	77.147	0	0
2 term models	2*3	43.15	0.01	0.01
	1*2	43.824	0.01	0.01
	2*4	45.944	0	0
	1*4	59.234	0	0
	1*3	60.449	0	0
	3*4	66.381	0	0
3 term models	2*3*4	34.722	1	0.68
	1*2*3	39.013	0.12	0.08
	1*2*4	44.604	0.01	0
	1*3*4	61.205	0	0
4 term models	1*2*3*4	37	0.32	0.22

Terms for model are as follows 1) Soil texture class, 2) tillage system, 3) type and 4) average number of worms.

**Figure 6:**
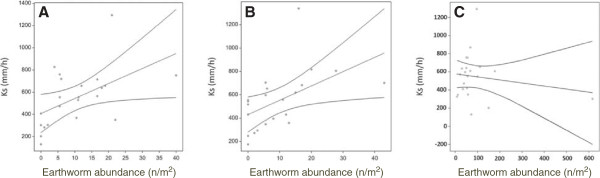
**Observed effect of earthworms on infiltration rate for abundance of (a) anecic earthworms, (b) abundance of epigeic earthworms, (c) abundance of endogeic earthworm.** Line show a linear regression fit, with dotted lines as 95% confidence intervals.

The relative contributions of different earthworm ecological groups (epigeic, endogeic and anecic) on infiltration rate were also assessed using data from those studies for which species identities were provided and their ecological classifications known. Within this restricted data-set of 22 relevant studies, a positive effect of earthworm numbers on infiltration rate was still found (p < 0.01). Comparing ecological groups, anecic and epigeic earthworms densities were both positively associated with increased infiltration (p = 0.05 and p = 0.03 respectively) (Figure 6a and b), while endogeic worm abundance had no effect on this metric (p > 0.05) (Figure 6c).

**Figure 7:**
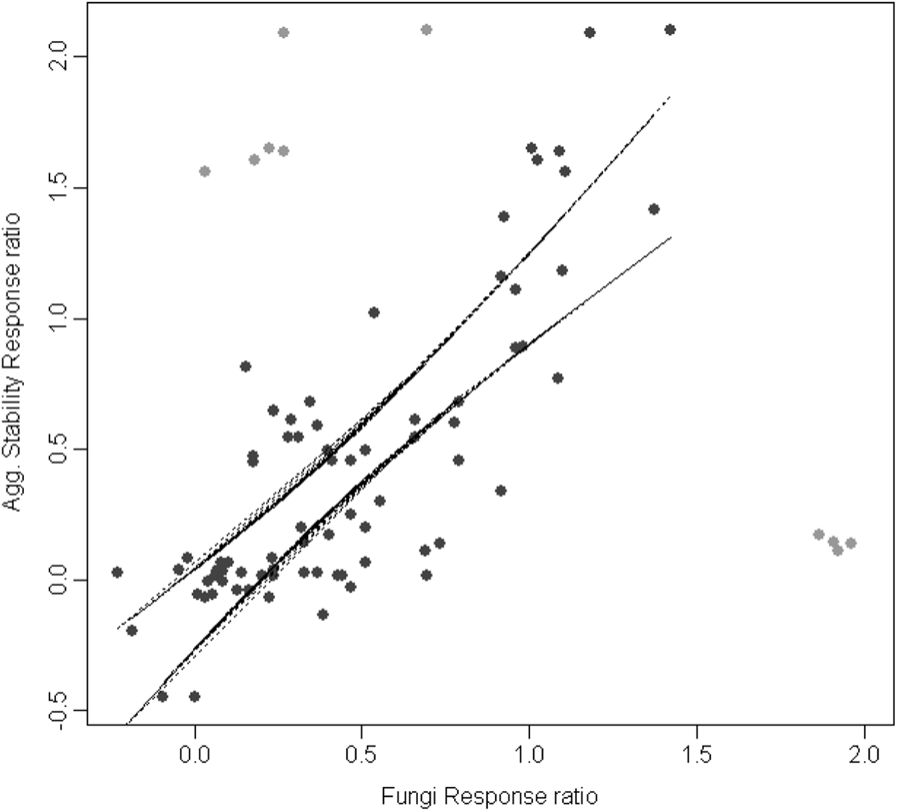
**Relationship between response ratios (Ln[×2/X’s]) of fungal biomass metrics and aggregate stability measures across land use transitions.** Light grey data-points were not included in the calculation of 95% confidence intervals (dashed lines) as assessed by Studentised residuals and Cooks distance.

For fungi and soil microaggregate stability, literature searching yielded 86 potential data sources from 10 papers (see Additional file 5). These produced data on response ratios for 41 tillage comparisons, 42 arable to grassland conversion and 3 grassland to woodland conversion. The fungal measure were glomalin-based in over 80% of these cases. Although some clear outliers were identified, a positive linear relationship between the responses to land use change in fungal biomass and soil microaggregate stability measures was indicated (Figure 7). Bootstrapping demonstrated that the model slope was accurate and significantly different from zero (Mean = 0.513; quantiles_0.05,0.95_ = 0.248, 0.894).

## Discussion and conclusions

Our meta-analyses for land-use transition effects on community metrics highlighted a consistent trend across the two studied taxa. This pattern relates declining land use intensity with positive effects on community parameters, especially in agricultural lands. This trend is consistent with a number of studies that have examined earthworm and fungal communities under different land uses at independent sites over larger spatial scales [10,20-24]. The value of our meta-analysis is that it provides a robust assessment of the effect sizes that are associated with steps in this transition. In the meta-analysis and in almost all individual studies, community metrics for both earthworms and fungi showed lowest values under intensive arable use. This is likely to be because soils under such management are subject to a combination of physical (e.g. tillage) and chemical (e.g. mineral fertiliser and pesticides) perturbations. When introduced, reduced or no-till management practices resulted in a generally significant positive effect on a range of community metrics for soil fungi. A similar significant positive effect of reducing tillage was also shown in a meta-analysis of primarily German data on tillage regime effects on earthworm abundance [25]. This latter study, however, also suggested that reduced tillage does not universally favour an increased density of all taxa, since Collembola numbers showed a trend for decrease under minimal management regimes.

The conversion of arable land to grassland represents a further reduction in land use intensity over reduced tillage, since soils under grassland are left to establish a normal depth profile. Further, the use of pesticides in such areas is often greatly reduced (compared to arable field) and organic inputs (dung, manures) can be greater. Conversion to pasture was found to be significantly beneficial for earthworm abundance and several fungal metrics. Analysis of density change with conversion time indicated a rapid accrual of earthworm numbers. Given the dominance of short-range dispersal in earthworms, the rapid change in earthworm densities following conversion, suggests that population increases are driven primarily by the recruitment of new progeny from the standing arable crop community. Since endogeic species may often be dominant in arable systems, it is such species that may be first to increase in number in response to grassland conversion. Later on as suitable food sources become available (especially surface litter) and recolonisation occurs, a more diverse community of epigeic, anecic and endogeic species can develop which can support or enhance ecosystem functions including water infiltration [26].

For fungi, a significant temporal trend in effect size of change in community metrics was seen following arable conversion to grassland. That effect sizes were positive across all major metric types, suggests an effect that is driven by abundance increases in a range of species from different fungal taxa. The general increase in fungal metrics seen is consistent with a series of studies that have reported such temporal effects. Thus in USA prairie lands, Bach et al. [27] found an asymptotic increase in arbuscular mycorrhizal fungal biomass between 0 and 18 years of restoration, while Van Der Wal et al. [22] found increases in fungal biomass and ergosterol across a chronosequence of abandoned arable land in the Netherlands. Such progressive changes in fungal community responses to grassland conversion suggest that colonisation rates may act to restrict the speed of fungal community change. However, there is also the potential for local controls on competition associated with the development of different organic substrates to regulate fungal community structure [28].

On grassland conversion to woodland, earthworm communities are generally maintained at previous grassland densities. For deciduous woodlands, identification of a consistent trend was challenging because of the high variation associated with effect size metrics. This variation probably reflects the strong effect of tree species composition on earthworm community development as observed in previous common garden experiments [29-31]. Only on conversion to coniferous woodland is there a suggestion of a negative impact on earthworm numbers (Figure 3). The relatively poor quality of coniferous needles as a food source [32], provides one explanation for this decline. Further, the chemistry and physical structure of soils associated with different woodlands may also affect soil habitat suitability for earthworms. For example, both earthworm survival and reproduction have been shown to be compromised in acidic soils [33,34]. However, a direct effect of pH change on effect size was not statistically supported by the meta-analysis.

For fungi, over time Rao et al. [35] found ectomycorrhizal infection and diversity to increase in a chronosequence of pine stands from 2 to 17 years old. In contrast, our analysis suggests an overall negative relationship with fungal measures and time since conversion, although in contrast to Rao et al. [35] this was for deciduous woodland (the only woodland type for which sufficient studies were available for a reliable analysis). Different patterns of response were noticeable between major metric types following woodland conversion providing insights into the nature of community change. Significant reductions in Biomass measures associated with increases in Colonisation measures suggests a shift to a community containing a greater proportion of mycorrhizal species, as would be expected in ecosystems dominated by trees. Further analysis of areas subject to afforestation are needed to fill gaps in knowledge concerning land use effects on fungi especially in non-deciduous systems and the resulting impacts for their ecological functioning.

An explicit aim of the current study was to go beyond providing quantitative information on the effects of land use change on earthworm abundance and fungal community metrics to assess also the potential effect of such change on the contribution to soil structure of the two studied taxa. Soil structure is dependent on features including the nature and stability of microaggregates and the development and maintenance of macropores that result from the burrowing activities of earthworms [36]. To date, however, the precise relationships between earthworm and fungal community metrics and soil structural properties have not been analysed systematically. For earthworms, constructions of regression models in this study to link relevant drivers to soil porosity using the data available mainly from field studies identified that earthworm abundance, tillage system and habitat type each had a significant effect on water infiltration rate.

More detailed analyses identified that the effects of individual earthworm ecological groups on infiltration differed. Anecic and epigeic earthworms increased water infiltration significantly, but this effect was not seen for endogeic worms. A positive effect was anticipated for the deep burrowing anecics, as they maintain vertical burrows for feeding and casting at the soil surface [26,37]. For this reason anecic earthworms have to date been the main ecological group considered in soil hydrological models [36]. Unlike anecics, epigeics are primarily surface dwellers living at the litter boundary or within this surface layer. Epigeics do not make vertical burrows, although shallow horizontal burrows can be maintained by some species [24,38,39]. Three factors relating to their lifestyle may explain the positive effect of epigeic species on water infiltration rates. Firstly, surface activity may prevent the formation of a soil surface crust of low water permeability. Secondly, the production and degradation of earthworm casts to form stable microaggregates can be important for soil moisture regulation and thus for water affinity and conductivity [40]. Finally, when exposed to adverse conditions (frost, drought), epigeic species may burrow deeper into the soil. At these times, epigeic earthworms can form temporary vertical burrows that may act as conduits for water flow.

The positive effects of anecic and epigeic species on water infiltration are important aspects for soil hydrology modelling. Bardgett et al. [41] outlined an approach that could be used to link earthworm community characteristics to soil hydrological processes. Both anecic and epigeic earthworms positively affect water transport to the deeper soil layer and ultimately to groundwater. This insight provides a challenge for land-management, since it is known that cultivation (disturbance) has a positive effect on soil hydraulic properties, yet such management is shown here to result in an average two to three fold reduction in earthworm abundance and negative effects on multiple soil fungal community metrics. Research to identify optimal strategies that exploit both the benefits of cultivation, while maintaining earthworm and fungal communities, is therefore needed to devise an approach that maximises hydrological processes to prevent run-off in managed lands.

Fungi too are known to contribute to soil structural development through hyphal growth and the production of coagulating substances like glomalin which may act as a ‘glue’ for soil microaggregates [15,16]. In this study, the positive relationship between the responses of fungi and soil microaggregate stability, which was generally consistent across studies and transitions, demonstrates a strong functional link between these microbes and soil structure properties. This reinforces similar positive relationships found between glomalin and aggregate stability at individual locations e.g. [16,42,43]. That glomalin levels in common with a number of other fungal community metrics respond positively to extensification as land-use transition from arable to pasture to woodland, suggests that measurement of this protein may be a useful integrative indicator of changes in the fungal community and associated soil functional metrics.

The wealth of species that are present in soils has long been recognised to contribute to many important ecological processes [44-46]. Quantifying precisely how land use and land management practices influence important soil taxa can provide practitioners with essential information that can be used to identify best practices for sustainable soil management. For example, in unmanaged systems understanding the contribution of earthworm and fungal communities to soil porosity and structure can provide useful information that can help to assess vulnerability to surface run-off and water logging. In managed systems, this detailed information can be used to identify best practice in relation to tillage levels and resulting effects on earthworms and fungi. The wider up-scaling of this knowledge through modelling to the landscape level can then be used to enhance the scientific basis for environmental economic modelling, providing a basis for sound decision making that optimises environmental benefits and enhance farmers’ income security [47].

## Methods

To provide quantitative data on the impact of land management and land use change on earthworm and fungal community metrics, an established systematic literature review optimised for the available project resources was followed [48,49]. Three clearly defined land management and land use transitions were considered. First, paired experiments in which fields within the same region were subjected to conventional tillage versus reduced/no till management (herein referred to as the “Tillage comparison”). No detailed results were presented here for earthworms because independent reviews of the effect of tillage on earthworm communities have recently been published [25,50], and also because a separate detailed meta-analysis of different tillage practices, soil type and sampling method effects on earthworm community size and diversity aspects is forthcoming (Schmidt et al., unpublished). Second, studies in which separate fields in a region have been kept under conventional arable use or converted to natural grassland or grazing pasture, or alternatively have been kept under arable versus grassland use for a known period (herein referred to as “Arable to grassland conversion”). Third, studies in which separate areas in a region have been kept as grassland or have undergone natural succession to tropical, deciduous or coniferous woodland or have been actively planted with trees for later agroforestry cropping (herein referred to as the “Grassland to woodland conversion”).

### Study identification and data collection

For the five community comparisons (two for earthworms, three for fungi) separate initial literature and short-listing search programmes were undertaken. Initially a search within the Web of Knowledge (Thomson Reuters) database was conducted in August 2011 to identify a set of potential references for detailed assessment. The search terms used for these searches were as follows. Earthworm populations for arable to grassland conversion - earthworm* *AND* (arable OR conversion OR crop* OR farmland OR grass* OR ley OR livestock OR rotation)*.* Earthworm populations for grassland to woodland conversion - earthworm* *AND* (abandonment OR afforestation OR agroforestry OR encroachment OR forest AND succession OR scrub OR shrub OR tree AND succession OR woodland OR woody). Fungal community for tillage to reduced tillage - (fung* OR arbuscular* OR mycorrhiza* OR saprotroph*) *AND* (*tillage OR no-till OR reduced-till*). Fungal community for arable to grassland conversion - (fung* OR arbuscular* OR mycorrhiza* OR saprotroph*) AND (arable OR conversion OR crop* OR farmland OR grass* OR grassland OR ley OR livestock OR grassland OR rotation). Fungal community for grassland to woodland conversion - (fung* OR arbuscular* OR mycorrhiza* OR saprotroph* AND (*abandonment OR afforestation OR agroforestry OR encroachment OR forest AND succession OR scrub OR shrub OR tree AND succession OR woodland OR woody*). Fungal community searches generally produced a higher number of hits than the corresponding earthworm searches. For the arable to grassland conversion search, the number of references identified for fungi was too high to be manageable for even an initial assessment (>10,000). In this case the terms were modified to exclude crop* and grass*. This produced a sub-set of 3,594 references for first pass assessment.

Once the initial reference list for the five community parameter searches had been collated, these reference details were reviewed by two scientists (D. Spurgeon, A. Keith). These reviews were done independently without consultation. During this initial review, each individual used the reference title and keywords to short-list papers that could feasibly contain relevant data. The criteria used for short-listing were that the study should 1) relate feasibly to the relevant taxa; 2) not be related solely to the results of a laboratory or mesocosm studies; 3) indicate investigation at the level of population or community (rather than biochemical or molecular studies); 4) not relate solely to studies of pollutant impacts on communities; 5) suggest that data relevant to the considered land-use or land management transitions may be included.

The two short-lists generated by the reviewers were then combined. For articles where both researchers identified from the title that the paper may contain relevant data, the full article was accessed and reviewed. All suitable data contained was collated into a single data resource for each meta-analysis. The information extracted included details of the literature source, geographical region of the study, site characteristic including time since transition and soil conditions (e.g. pH, soil organic matter content and texture) before and after transition, land management regimes including crop types and grazing regime, as well as the community metrics with error estimates (standard error, standard deviation) and replication. For those articles where only one researcher identified that the paper may contain relevant data, article abstracts were accessed and where feasible reviewed (earthworms - D. Spurgeon, fungi – A. Keith) to provide a greater degree of insight into the details of the study. If the abstract confirmed that the published article was consistent with the selection criteria, then the full article was reviewed and all appropriate information and data incorporated into the database.

The analysis to link earthworm community metrics to soil hydrological properties followed a broadly similar approach to the five land use change and community size assessments described above. For the earthworm study, searches were conducted in July 2011 in Google scholar using keywords: “*soil water infiltration*” AND “earthworm*”. Infiltration rate as the velocity of water entering into soil (mm h^-1^) was used as the assessed parameter, and the equilibrium infiltration rate, which is the steady state infiltration rate which nearly equals the saturated hydraulic conductivity of soil (*Ks*), was the measurement parameter. These searches generated 174 hits. Hence, in this case it was possible for two researchers (J. Faber, D. Lammertsma) to review the abstract of each article jointly to confirm the potential to contain relevant data based on criteria that the study 1) related to earthworms; 2) included assessment in soil under field or mesocosm conditions; 3) suggested inclusion of physical measures of soil hydrological properties. Short-listing was followed by full article review and data-extraction where appropriate. Articles on (semi)arid and tropical systems were omitted. For the analysis linking fungal and soil structural measures, the papers identified in the literature searches for fungi were assessed to find those containing suitable data on both fungal biomass and soil microaggregate stability measures. These were augmented with further papers identified in a WoK search using the keywords: *“aggregate stability” AND “fungi”*. In almost all cases, the measures of aggregate stability given in the analysed paper based on the quantity of water stable soil microaggregates of size >0.25 mm (see Additional file 4).

### Data handling

Where appropriate data were pooled across different depths. Given the variety of metrics, we used Hedges’ G standardized effect size (ES) to estimate the influence of different land use transitions on both earthworm and fungal community measures. A high level of heterogeneity was found within the data-sets resulting from the range of experiment types and systems addressed as well as measures used for fungi. This heterogeneity meant that synthesis based approaches, such as forest plot analysis, were not appropriate. Instead, for assessing the drivers of community effects, standard quantitative and meta-analysis statistics including vote counting, fixed effect and mixed model analysis and linear and non-linear regression were used. For the analysis of earthworm effects on infiltration rate, we used GLM with a normal link function. The best model was selected using all subsets regression on basis of Akaike weights information. The relationship between the response ratios (Ln [×2/X’s]) of fungal biomass and soil microaggregate stability measures was tested using a linear model. Outliers and influential data-points were assessed using studentised residuals and Cooks distance. Since model assumptions could not be satisfied, a mean model slope was generated by bootstrapping and quantiles used to assess its significance. These analyses were conducted in the R statistical environment [51].

### Availability of supporting data

Data-sets relating to these meta-analyses have been included as separate supplementary files (as Microsoft Excel or comma separated text) that accompany this manuscript.

## Competing interest

The authors confirm that they have no competing financial or non-financial interests.

## Authors’ contributions

DJS conducted references searches, data compilation and analysis for the earthworm abundance meta-analyses; AMK conducted the references searches, data compilation and analysis for the fungal meta-analyses; OS provided information and data for tillage effect meta-analyses and DRL and JHF undertook the functional meta-analyses for earthworms. All authors contributed to the writing. All authors read and approved the final manuscript.

## Supplementary Material

Additional file 1 Meta-data codes for four meta-analysis data-sets on the effects of land use change on earthworm and fungal community parameters, earthworm community parameter effects on soil hydrological properties and fungal community parameters on soil aggregate stability. Click here for file

Additional file 2 Meta-analysis data-sets for the effects of land management and land-use changes on earthworm abundance. Click here for file

Additional file 3 Meta-analysis data-sets for the effects of land management and land-use changes on soil fungal community parameters. Click here for file

Additional file 4 Meta-analysis data-sets for the relationship between earthworm community parameters and soil hydrological properties. Click here for file

Additional file 5 Meta-analysis data-sets for the relationship between soil fungal community characteristics and soil micro-aggregate stability. Click here for file
