# Supplementary material for: Land-use and land-management change: relationships with earthworm and fungi communities and soil structural properties
Source: BMC Ecol. 2013 Dec 1;13:46. doi: 10.1186/1472-6785-13-46 (PMC4219611; doi:10.1186/1472-6785-13-46)
Supplement: Additional file 1 — Meta-data codes for four meta-analysis data-sets on the effects of land use change on earthworm and fungal community parameters, earthworm community parameter effects on soil hydrological properties and fungal community parameters on soil aggregate stability. [file 1472-6785-13-46-S1.docx]

**Title: Meta-analysis data-sets for the effects of land management and land-use changes on earthworm abundance**

This meta-analysis data-set was conducted within the EU EcoFinders project using a traditional meta-analysis approach involving systematic literature searches followed by progressive screen and review of identified papers for ultimate data extraction. Such quantitative literature assessments were to quantify the effects of land use and land management change on earthworm and soil fungal communities for earthworms across a range of successional/extensification transitions (conventional arable → no or reduced tillage → grassland → wooded land). Two scenarios were considered for their effects on earthworm counts namely, 1) conversion of managed arable land to natural or managed grassland; 2) successional conversion or afforestation of grassland to woodland. Three scenarios were considered for their effects on fungi counts namely, 1) transfer of soil tillage regime from conventional to conservation tillage or no-till management, 2) conversion of managed arable land to natural or managed grassland; 3) successional conversion or afforestation of grassland to woodland. The data-sets of the effects of earthworm diversity relate the abundance of earthworms to the saturated hydraulic conductivity of soil (mm/h). The data-sets for the relationship between fungal community and soil micro-aggregate stability relate fungal biomass and protein measurements to water stable and crumb aggregate stability measurements.

Spurgeon, D.J., Keith, A. M. Schmidt, O., Lammertsma, D., Faber, J. H. (2013). Land use change and management effects on soil diversity and regulation of water flows in soil. *BMC Ecol.*

**Metadata Earthworm Abundance**

**Format of Stored Data:** Spreadsheets in CSV format

**Description of headers**

| **Header** | **Description** |
| --- | --- |
| Land-use conversion | Description of the land use change case represented in literature study |
| Study Country | Location country of the reported earthworm community study. |
| Climate region | Major climatic region of the study country of the reported study |
| Reported rainfall for study region stated in paper | Rainfall in millimetres reported for the sites at which earthworm communities were assessed |
| Initial land-use soil percent clay | Percentage clay fraction in the soils under the initial land-use (i.e. prior to change) |
| Final land-use soil percent clay | Percentage clay fraction in the soils under the initial final-use (i.e. after change) |
| Initial land-use soil percent organic matter | Percentage organic matter in the soils under the initial land-use (i.e. prior to change) |
| Final land-use soil percent organic matter | Percentage organic matter in the soils under the initial final-use (i.e. after change) |
| Initial land-use soil pH | Soil pH in the soils under the initial land-use (i.e. prior to change) |
| Final land-use soil pH | Soil pH in the soils under the initial final-use (i.e. after change) |
| Units of pH shift on initial to final land-use change | Difference in pH (units) in soils measured before and subsequent to land use change after the number years specified in the study |
| Stated arable crop type | Crop regime in the arable field situation prior to grassland conversion |
| Is pasture grazed/productive | Grazing status (yes/no) of the pasture at sites measured after conversion to grassland. |
| Evidence for grazing status | Evidence given in the literature reference to support the designation of grazing status of the pasture at sites after conversion to grassland. |
| Stated forest type | Major forest class of the reforested woodlands developing after re-growth from pasture |
| Stated years since land use change | Stated range in years for the sites sampled for earthworm abundance to assess effects of land use change |
| Stated years since land use change minimum | The minimum number in years for the sites sampled for earthworm abundance to assess effects of land use change |
| Stated years since land use change class | Classification of study to 5 classes based on the time elapsed since land-use conversion (1=0-3 years, 2=4-9 years, 3=10-20 years, 4=21-50 years, =>50 years). |
| Data Source in paper | Source of data (Table, Figure) used to provide the quantitative data on earthworm counts for metal-analysis. |
| Initial land-use mean earthworm abundance | Mean abundance of earthworm (per metre square) collected in sites under the initial land-use. |
| Initial land-use standard deviation for earthworm abundance | Standard deviation of earthworm (per metre square) collected in sites under the initial land-use. |
| Initial land-use standard error for earthworm abundance | Standard error of earthworm (per metre square) collected in sites under the initial land-use. |
| Initial land-use number of replicates | Total number of replicate samples taken to assess earthworm abundance (per metre square) collected in sites under the initial land-use. |
| Final land-use mean earthworm abundance | Mean abundance of earthworm (per metre square) collected in sites under the final land-use. |
| Final land-use standard deviation for earthworm abundance | Standard deviation of earthworm (per metre square) collected in sites under the final land-use. |
| Final land-use standard error for earthworm abundance | Standard error of earthworm (per metre square) collected in sites under the final land-use. |
| Final land-use number of replicates | Total number of replicate samples taken to assess earthworm abundance (per metre square) collected in sites under the final land-use. |
| Primary reference source | Full reference for the collated data-set |

**Metadata Fungal Communities**

**Format of Stored Data:** Spreadsheets in CSV format

Description of headers

| **Header** | **Description** |
| --- | --- |
| **Land-use conversion** | Description of the land use change represented in study |
| **Study_code** | Unique code within land use conversion category assigned to primary reference source. |
| **Country** | Country in which the study was conducted. |
| **Location** | Location at which the study was conducted. |
| **Koeppen_climate** | Major climate group of Koeppen classification associated with location. |
| **Koeppen_code** | Code of Koeppen classification associated with location. |
| **Mean_Annual_Precipitation** | Mean annual precipitation (mm) in region; taken from online sources if not reported and available. |
| **Soil_texture** | Soil texture at study location reported in primary reference source. |
| **Sample_year** | Year in which sampling was conducted |
| **Sample_month** | Month in which sampling was conducted |
| **Sample_depth** | The depth range of soil in which fungal metrics were assessed. |
| **Metric_type1** | Broad categories of fungal metrics i.e. Biomass, Colonisation or Diversity |
| **Metric_type2** | Specific types within the broad categories of fungal metrics e.g. PLFA, Glomalin, Root length. |
| **Metric_units** | Measurement units of fungal metrics. |
| **Metric_notes** | Further details of methods or fungal metrics. |
| **Transition_type** | Change (Prior management and transition time defined) OR Comparison (Prior management of second land use or transition time not defined). |
| **Transition_description** | Description of initial and final land use taken from primary reference source |
| **Transition_notes** | Further details of initial and final land use taken from primary reference source. |
| **LU1_year** | Year that initial land-use was started. |
| **LU2_year** | Year that final land-use was started. |
| **Year_of_change** | Year when land-use change occurred. |
| **Years_since_change** | Number of years since land-use change occurred. |
| **Crop_sampled** | Crop regime in the arable system at sampling. |
| **Crop_other** | Other crops grown in arable system prior to sampling. |
| **Crop_tillage** | Tillage regime in arable system i.e. conventional, reduced till or no till. |
| **Is pasture grazed?** | Grazing status (yes/no) of the pasture at sites measured after conversion to grassland. |
| **Forest class** | Major forest class of the woodlands developing on natural or managed grassland. |
| **Forest species** | Main tree species or tree types developing on natural or managed grassland. |
| **LU1_OM** | Percentage organic matter in the soils under the initial land-use (i.e. prior to change) |
| **LU2_OM** | Percentage organic matter in the soils under the initial final-use (i.e. after change) |
| **LU1_ph** | Soil pH in the soils under the initial land-use (i.e. prior to change) |
| **LU2_ph** | Soil pH in the soils under the initial final-use (i.e. after change) |
| **Data_source_in_paper** | Source in primary reference from which data is derived. |
| **Source_ES** | 1=Means & SD, 2=F-statistic (1 df), 3=P-value, 4= Pooled SD derived from LSD, 5=Assumed zero from reported null effect. |
| **Source_means** | 1=Raw data (e.g. from Supplementary data or directly from author), 2=Directly reported, 3=Obtained from graph, 4=Average of reported means. |
| **Source_SD** | 1=Raw data (e.g. from Supplementary data or directly from author), 2=Directly reported, 3=Obtained from graph, 4=Pooled from reported variance, 5=Pooled SD calculated. |
| **LU1_N** | Total number of replicate samples taken to assess fungal metrics under the initial land-use. |
| **LU2_N** | Total number of replicate samples taken to assess fungal metrics under the final land-use. |
| **LU1_mean** | Mean value of fungal metrics under the initial land-use. |
| **LU2_mean** | Mean value of fungal metrics under the final land-use. |
| **LU1_sd** | Standard deviation of fungal metrics under the initial land-use. |
| **LU2_sd** | Standard deviation of fungal metrics under the final land-use. |
| **Primary reference source** | Full reference for the collated data-set |

**Metadata Earthworm communities and soil hydrology**

**Format of Stored Data:** Spreadsheets in CSV format

**Description of headers**

| **Header** | **Description** |
| --- | --- |
| Author | Lead author reference and publication year |
| Country | Location country of the reported study |
| Plot description | Description of the sample plot used for the study |
| Sampling method | Method used for the collection of earthworms for community assessment |
| Average Number of worms | Mean abundance of earthworm (per metre square) collected in the field site |
| Number of plots | Total number of replicate samples taken to assess earthworm abundance (per metre square) collected in the study sites |
| Average Number of epigeics | Mean abundance of epigeic earthworm (per metre square) collected in the field site |
| Average Number of endogeics | Mean abundance of endogeic earthworm (per metre square) collected in the field site |
| Average Number of anecics | Mean anecic of epigeic earthworm (per metre square) collected in the field site |
| Saturated hydraulic conductivity of soil (mm/h) | Measurement of the saturated hydraulic conductivity of the soil which described water movement through saturated media |
| Number of plots | Total number of replicate samples taken to saturated hydraulic conductivity of the soil collected in the study sites |
| Soil texture class | Soil texture in the field sites used for study |
| Habitat type | Habitat type in the field sites used for study |
| Tillage system | Main tillage regime |

**Soil fungi and micro-aggregate stability**

**Format of Stored Data:** Spreadsheets in CSV format

Description of headers

| **Header** | **Description** |
| --- | --- |
| Land-use conversion | Description of the land use change case represented in literature study |
| LU1_description | Description of initial land use taken from primary reference source |
| LU2_description | Description of final land use taken from primary reference source |
| Years since conversion | Number of years since land-use change |
| Fungal metric type | Type of metric used to assess fungal biomass |
| Fungal metric | Metric used to assess fungal biomass |
| Aggregate stability measure | Measure used to assess aggregate stability |
| LU1_fungal_mean | Mean value of fungal metric under initial land use |
| LU2_fungal_mean | Mean value of fungal metric under final land use |
| LU1_aggregate_stability_mean | Mean value of aggregate stability measure under initial land use |
| LU2_aggregate_stability_mean | Mean value of aggregate stability measure under final land use |
| Odds_ratio_fungal | Log odds ratio of fungal metrics in initial and final land uses |
| Odds_ratio_aggregate_stability | Log odds ratio of aggregate stability measures in initial and final land uses |
| Primary reference source | Full reference for the collated data-set |
